# Enhancement of the SESN2-SHP cascade by melatonin ameliorates hepatic gluconeogenesis by inhibiting the CRBN-BTG2-CREBH signaling pathway

**DOI:** 10.1038/s12276-023-01040-x

**Published:** 2023-07-24

**Authors:** Seungwon An, Balachandar Nedumaran, Hong Koh, Dong Jin Joo, Hyungjo Lee, Chul-Seung Park, Robert A. Harris, Keong Sub Shin, Ali R. Djalilian, Yong Deuk Kim

**Affiliations:** 1 Department of Ophthalmology and Visual Sciences, University of Illinois at Chicago, Chicago, IL 60612 USA; 2 Barbara Davis Center for Diabetes, School of Medicine, University of Colorado Anschutz Medical Campus, Aurora, CO 80045 USA; 3 Division of Gastroenterology, Hepatology and Nutrition, Department of Pediatrics, Severance Children’s Hospital, Severance Pediatric Liver Disease Research Group, Yonsei University College of Medicine, Seoul, 03722 Republic of Korea; 4 Department of Surgery, Yonsei University College of Medicine, Seoul, 03722 Republic of Korea; 5 School of Life Sciences and Cell Logistics Research Center, Gwangju Institute Science and Technology, Gwangju, 61005 Republic of Korea; 6 Department of Biochemistry and Molecular Biology, Indiana University School of Medicine, Indianapolis, IN 46202 USA; 7 DUKSAN Institute of Biomedical and Life Science, Gwangmyeong, 14348 Republic of Korea; 8 Young Sciences, Inc., Bucheon, 14449 Republic of Korea; 9 Research Institute of Aging and Metabolism, Kyungpook National University, Daegu, 41566 Republic of Korea

**Keywords:** Type 2 diabetes, Metabolic syndrome

## Abstract

Melatonin is involved in the regulation of various biological functions. Here, we explored a novel molecular mechanism by which the melatonin-induced sestrin2 (SESN2)-small heterodimer partner (SHP) signaling pathway protects against fasting- and diabetes-mediated hepatic glucose metabolism. Various key gene expression analyses were performed and multiple metabolic changes were assessed in liver specimens and primary hepatocytes of mice and human participants. The expression of the hepatic cereblon (CRBN) and b-cell translocation gene 2 (BTG2) genes was significantly increased in fasting mice, diabetic mice, and patients with diabetes. Overexpression of *Crbn* and *Btg2* increased hepatic gluconeogenesis by enhancing cyclic adenosine monophosphate (cAMP)-responsive element-binding protein H (CREBH), whereas this phenomenon was prominently ablated in *Crbn* null mice and *Btg2*-silenced mice. Interestingly, melatonin-induced SESN2 and SHP markedly reduced hepatic glucose metabolism in diabetic mice and primary hepatocytes, and this protective effect of melatonin was strikingly reversed by silencing *Sesn2* and *Shp*. Finally, the melatonin-induced SESN2-SHP signaling pathway inhibited CRBN- and BTG2-mediated hepatic gluconeogenic gene transcription via the competition of BTG2 and the interaction of CREBH. Mitigation of the CRBN-BTG2-CREBH axis by the melatonin-SESN2-SHP signaling network may provide a novel therapeutic strategy to treat metabolic dysfunction due to diabetes.

## Diabetes: Signalling pathway interactions regulated by melatonin

The identification of a fundamental signalling process regulated by melatonin that can prevent excessive liver glucose metabolism during diabetes may offer a novel therapeutic approach. Both fasting and diabetic states trigger the elevation of a particular signalling pathway that significantly increases liver glucose metabolism, or ‘hepatic gluconeogenesis’, resulting in a rise in blood sugar levels. In experiments on mouse models and human patients, Yong Deuk Kim at Kyungpook National University in Daegu, South Korea, and co-workers have demonstrated that the hormone melatonin plays a vital role in suppressing hepatic gluconeogenesis. Melatonin upregulates another signaling cascade involving a stress regulation protein called sestrin-2 and its associated regulatory protein, SHP. The upregulation of SHP by melatonin repressed genes that are involved in gluconeogenesis, both in diabetic mice and in the human liver.

## Introduction

Melatonin is produced by the pineal gland and is involved in the regulation of diverse physiological processes, such as anti-inflammatory, antioxidant, lipid metabolic, and glucose metabolic processes^1–3^. Melatonin coordinates several molecular factors, including sirtuin 1 (SIRT1), peroxisome proliferator-activated receptor gamma-coactivator-1α, nuclear respiratory factor-1, transcription factor A, retinoid orphan receptor, and noncoding RNAs^4–8^. Moreover, the melatonin-AKT signaling pathway suppresses hepatic gluconeogenesis via melatonin receptors (MT1/MT2) in rodent models^9^. Melatonin prevents sepsis-induced liver injury and dysregulation of hepatic gluconeogenesis by targeting SIRT1-signal transducer and activator of transcription 3 (STAT3) activation in the liver^10^. However, the absence of melatonin prompts hepatic gluconeogenesis by stimulating the unfolded protein response in rats with pinealectomy^11^.

Cereblon (CRBN) is primarily expressed in diverse tissues and is involved in the regulation of metabolic disturbance by downregulating AMP-activated protein kinase (AMPK)^12–14^. Intriguingly, *Crbn*-deficient mice exhibit hyperphosphorylated AMPK and improved insulin sensitivity^15^. B-cell translocation gene 2 (BTG2) is a key regulator of various cellular processes, including cell growth, differentiation, and apoptosis. This molecule is induced by hypoxia, oxidative stress, and metabolic changes and inhibited by insulin and growth factors^16,17^. Previous studies have shown that BTG2 regulates hepatic gluconeogenic gene transcription by glucagon-CREB signaling and/or Nur77 interaction^18,19^. Cyclic adenosine monophosphate (cAMP)-responsive element-binding protein H (CREBH) is a basic leucine zipper transcription factor and is highly expressed in diverse tissues, such as the liver, skeletal muscle, and stomach^20^. CREBH is induced by fasting, glucocorticoid receptor, proinflammatory cytokines, and endoplasmic reticulum (ER) stress and is repressed by insulin. CREBH plays a crucial role in regulating glucose and lipid metabolism by interacting with several nuclear receptors and transcription factors^21^. However, the potential role of the hepatic CRBN-BTG2-CREBH axis during fasting- and diabetes-mediated glucose metabolism remains largely unexplored.

Sestrin2 (SESN2) is induced by pathophysiological events, such as oxidative stress, hypoxia, and nutritional stimuli. SESN2 has a beneficial effect on physiological processes, including obesity, insulin resistance, and inflammation^22^. Loss of SESN2 is known to increase oxidative stress and ER stress, which can lead to liver damage and fibrosis^23^. Small heterodimer partner (SHP; NR0B2) is an orphan member of the nuclear receptor superfamily and lacks the classical DNA-binding domain. SHP functions as a transcriptional corepressor by directly interacting with several nuclear receptors and transcription factors. SHP has a vital role in the maintenance of metabolic substances, including glucose, lipids, bile acid metabolism, and inflammation^24,25^. However, the potent role of the SESN2-SHP signaling pathway in hepatic gluconeogenesis and its subsequent role remain unknown.

Herein, we demonstrate that elevation of the CRBN-BTG2-CREBH signaling pathway is induced by fasting and diabetic conditions. CRBN and BTG2 significantly increase hepatic glucose metabolism via the induction of CREBH in diabetic mice and primary hepatocytes. Melatonin ameliorates fasting- and diabetes-mediated induction of hepatic gluconeogenesis via the stimulation of the SESN2-SHP cascade. Overall, our findings suggest that the melatonin-SESN2-SHP signaling network provides a strong basic molecular mechanism. This study will be preclinically and/or therapeutically important to target one or more of our identified members to alleviate hepatic metabolic dysfunction under fasting and diabetic conditions.

## Materials and methods

### Human subjects

Human liver samples were collected at Yonsei University College of Medicine (Republic of Korea) following the Institutional Review Board approved study protocol (No. 2019-0595-016, 2021-2931-001). Liver biopsy from healthy donors (NOR, *n* = 5) and patients diagnosed with diabetes mellitus (DM, *n* = 6) was performed with consent. All protocols were approved by the Medical Ethics and Clinical Research Committee following the guidelines and policies of the Declaration of Helsinki.

### Animals

Male wild-type C57BL/6 J (Samtako BioKorea, Seoul, Republic of Korea) mice and CRBN null mice were used as described previously^15^. For fasting and feeding condition studies, the mice were fed *ad libitum* and fasted for 9 h. WT and streptozotocin (STZ, 80 mg/kg body weight, Sigma‒Aldrich, St. Louis, MO, USA)-induced diabetic mice were treated for 14 days^19^. WT and CRBN null mice were fed a chow diet (CD) and a high-fat diet (HFD) as mentioned previously^15^. WT and *db*/*db* mice were administered melatonin (Sigma‒Aldrich, 20 mg/kg of body weight) once daily for 14 days. For viral transduction, adenoviruses expressing *Crbn*, *Btg2*, *Sesn2* (*Se2*), and *Shp* (1 × 10^9^ plaque-forming units) were intravenously injected into WT and *db*/*db* mice. For the silencing of *Btg2*, lentivirus expressing sh*Btg2* (1 × 10^9^ transducing units/mL) was intravenously injected into *db*/*db* mice. For silencing viral transduction, WT and *db*/*db* mice were injected with Ad-sh*Crbn*, Ad-sh*Sesn2* (sh*Se2*), Ad-small interfering (si)RNA *Crebh*, and Ad-si*Shp* (1 × 10^9^ plaque-forming units) via tail vein injection. Total RNA was extracted from the livers of adenovirus- and lentivirus-infected mice for quantitative real-time polymerase chain reaction (qPCR) analysis. Blood glucose levels of all mice were measured with Glucostix Accu-Check (Roche Diagnostics, Mannheim, Germany). Animal experiments were approved by the Institutional Animal Care and Use Committee of the University of Illinois at Chicago (#20-079) and Raon Bio (KPCP-140029) according to the policy of the National Institutes of Health.

### Measurement of metabolic parameters

Plasma glucose was measured using Glucostix Accu-Check (Roche Diagnostics, Mannheim, Germany) as previously described^19,26^. For glucose tolerance tests, the mice were fasted for 14 h and then intraperitoneally injected with 1 g/kg glucose. For insulin tolerance tests, the mice were fasted for 4 h and intraperitoneally injected with 0.5 units/kg insulin. The blood glucose levels were measured under the indicated conditions after each injection.

### RNA extraction and qPCR analysis

Total RNA was isolated using the TRIzol method (Invitrogen, Carlsbad, CA, USA), as mentioned previously^19^. mRNA expression was determined using the Power SYBR^®^ Green PCR Master Mix kit (Applied Biosystems, Warrington, UK) and the StepOne^TM^ Real-time PCR system (Applied Biosystems) for qPCR analysis. We utilized gene-specific primers for measuring the *Crbn, Btg2, Crebh, Pck1, G6pc, Sesn2*, and *Shp* genes by qPCR^15,19,27,28^. Gene expression was normalized to ribosomal L32 expression.

### Immunoblotting

Proteins were collected from each sample and analyzed according to the methods described previously^19,28^. BTG2, CREBH, SHP, pAKT, total AKT, β-actin (Santa Cruz Biotechnology, Santa Cruz, CA, USA), CRBN, pAMPK, AMPK, pS6K, S6K (Cell Signaling Technology, Danvers, MA, USA), and SESN2 (Protein Tech, Chicago, IL, USA) antibodies were used as described previously^9,14,19,27–30^.

### Culture of primary hepatocytes and glucose output assay

Primary hepatocytes were isolated from the livers of male 8-week-old mice using a portal vein collagenase (Sigma‒Aldrich) perfusion method as described previously^19^. Primary mouse hepatocytes were maintained in M199 medium (Cellgro, Herdon, VA, USA). The cells were utilized for adenoviral infection and FSK (10 μM) or melatonin (500 μM) treatment. Glucose production was performed using a glucose oxidase assay kit (Sigma‒Aldrich) as mentioned previously^19^.

### Plasmids, cell culture, and transient transfections

The reporter plasmid containing the *Pck1* and *G6pc* promoters was previously described^19^. The *Crebh*, *Btg2*, and *Shp* expression vectors were previously described^19,27,28^. AML-12 cells were cultured in Dulbecco’s modified Eagle’s medium (DMEM)/F-12 medium (Gibco-BRL, Grand Island, NY, USA) with 10% fetal bovine serum (FBS, HyClone, Logan, UT, USA), 1% insulin-transferrin-selenium mixture (ITS, Gibco-BRL), dexamethasone (40 ng/ml, Sigma‒Aldrich), and 1% antibiotics (Gibco-BRL). The cells were maintained in a humidified atmosphere containing 5% CO_2_ at 37 °C. Transient transfection assays were conducted using AML-12 cells, as previously described^19^. Briefly, transient transfection was performed using Lipofectamine 2000 (Invitrogen, Carlsbad, CA, USA) according to the manufacturer’s protocol. Cytomegalovirus-β-galactosidase plasmids were used as an internal standard to adjust for transient transfection efficiency.

### Recombinant adenoviruses

Adenoviruses harboring *Crbn*, *Btg2*, *Crebh*, *Shp*, Ad-si*Crebh*, Ad-si*Shp*, and green fluorescent protein (GFP) were generated as previously described^19,27–29^. Ad-*Sesn2* (Ad-*Se2*) and Ad-sh*Sesn2* (Ad-sh*Se2*) were purchased from Vector Biolabs (Malvern, PA, USA). A recombinant lentiviral delivery system (Dharmacon, Lafayette, CO, USA) of *Btg2*-targeted short hairpin (sh)RNA (sh*Btg2*) was prepared as previously described^19^.

### Coimmunoprecipitation (IP) assay

Total liver protein extracts were subjected to immunoprecipitation with antibodies (SHP and CRBH; Santa Cruz Biotechnology). The immunoprecipitated proteins were immunoblotted with specific antibodies as previously described^27,29^. Membranes were developed using an Amersham Detection kit (GE Healthcare, Piscataway, NJ, USA).

### Chromatin immunoprecipitation (ChIP)

The ChIP assay was carried out with corresponding antibodies according to the manufacturer’s protocol (Upstate Biotechnology, Upstate, NY, USA), as previously described^19,29^. Briefly, crosslinked lysates were prepared with the corresponding beads and salmon sperm DNA. The purified DNA extractions were quantified by qPCR using the proximal region of the *Pck1* promoter (F: 5′-CAGACTTTGTCTAGAAGTTT-3′, R: 5′-TCTTGCCTT-AATTGTCAGGT-3′) and nonspecific distal region of the *Pck1* promoter (F: 5′-TGCCATGG-CTCACAGTGCCT-3′, R: 5′-GTTACGAAATGACCTGGAGG-3′).

### Statistical analysis

Statistical calculations were conducted using GraphPad Prism software (GraphPad, USA). Statistical significance was determined using two-tailed Student’s *t* test or one-way analysis of variance. All data are presented as the mean±SEM. A *p* < 0.05 was considered statistically significant.

## Results

### CRBN, CREBH, and BTG2 gene expression in the liver specimens of diabetic mice and human patients with DM

**Fig. 1:**
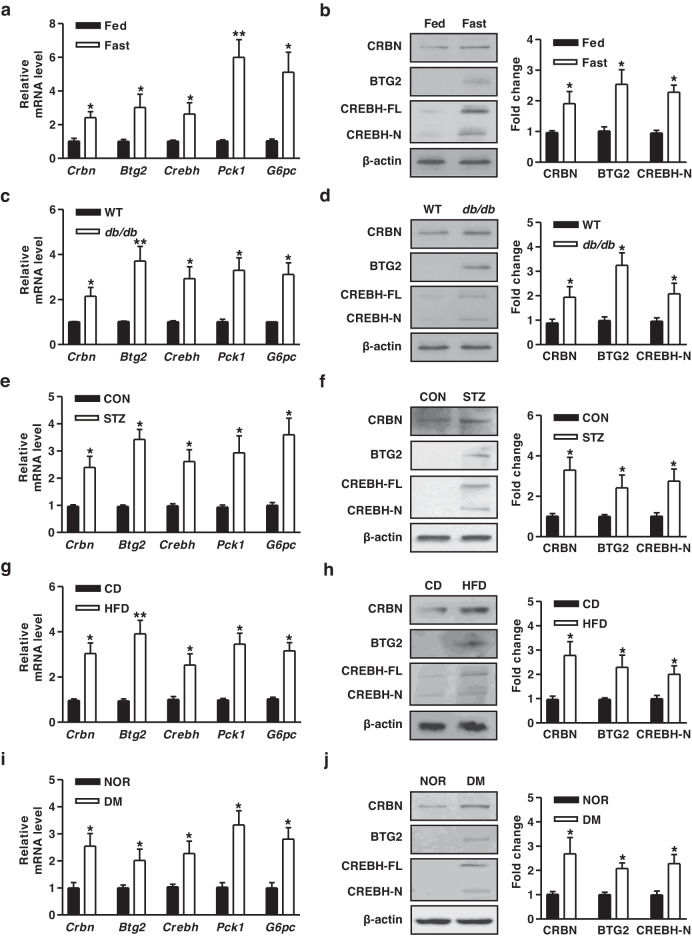
Expression of *Crbn, Crebh*, and *Btg2* genes in the liver specimens of diabetic mice and diabetic patients. **a, b** WT mice were fed *ad libitum* (Fed) and fasted for 9 hr (Fast). **a** Gene expression analysis using gene-specific qPCR primers. **b** Western blot analysis using the indicated antibodies. **c, d** WT and *db*/*db* mice were fasted for 9 hr. **c** Gene expression analysis. **d** Western blot analysis (FL, full-length; N, nuclear). **e** qPCR analysis of total RNA from the livers of control (CON) mice and streptozotocin (STZ, 80 mg/kg)-induced diabetic mice for 14 days. **f** Various protein levels in liver tissue extracts were analyzed by immunoblotting using specific antibodies. **g** qPCR analysis of total RNA from the livers of chow diet (CD) and high-fat diet (HFD) mice for 14 weeks. **h** Tissue extracts were analyzed by immunoblotting with the indicated antibodies. *n* = 4-5 mice per group. **i** qPCR analysis was performed using liver biopsies from healthy donors (NOR) and diabetes mellitus (DM) patients using the indicated primers. **j** Tissue extracts were analyzed by immunoblotting with specific antibodies. *n* = 5-6 per group. ^*^P < 0.05, ^**^P < 0.01vs. fed mice, WT mice, CON mice, CD mice, or healthy donors.

We investigated the expression of characteristic genes during prolonged fasting in mice. Mice with prolonged fasting exhibited enhanced expression of the CRBN, BTG2, and CREBH genes and hepatic gluconeogenic genes (*Pck1*, *G6pc*) compared to fed mice (Fig. 1a, b). Similarly, increases in the mRNA and protein levels of *Crbn*, *Btg2*, *Crebh*, and hepatic gluconeogenic genes were induced in *db*/*db* mice (Fig. 1c, d). We also examined these genes in other diabetic mouse models, including streptozotocin (STZ)- and high-fat diet (HFD)-induced diabetic mice. These genes were significantly increased in STZ- and HFD-induced diabetic mice (Fig. 1e–h). To further evaluate the biological consequence of these genes in human participants with diabetes mellitus (DM), we performed qPCR and immunoblot analysis using liver specimens from healthy donors (NOR) and DM patients. As shown in Fig. 1i, j, the expression of key metabolic genes was considerably higher in the livers of DM patients than in those of healthy donors. Taken together, these findings suggest that the hepatic CRBN, CREBH, and BTG2 genes are upregulated during fasting and in patients with diabetes.

### CRBN induces hepatic gluconeogenesis

**Fig. 2:**
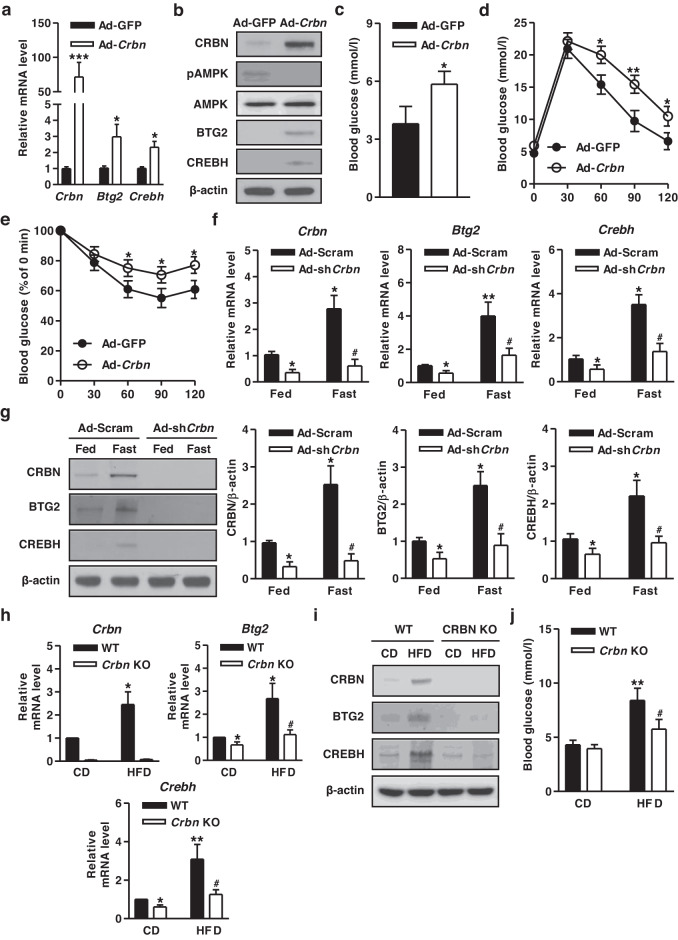
CRBN induces hepatic gluconeogenesis. **a–e** WT mice were injected with Ad-GFP and Ad-*Crbn* via the tail vein for 10 days. **a** Gene expression analysis using various gene-specific primers. **b** Immunoblotting with specific antibodies. **c** Blood glucose levels on Day 10 in Ad-*Crbn*-infected mice. **d** Glucose tolerance test in the indicated groups at Day 4 after adenoviral infection. **e** Insulin tolerance test at Day 8 after adenoviral infection. **f, g** WT mice were injected with Ad-Scramble (Ad-Scram) and Ad-sh*Crbn* via the tail vein for 10 days. After injection, these mice were fed *ad libitum* (fed) and fasted for 9 h (fast). **f** Total RNA was utilized for qPCR analysis with gene-specific primers. **g** Tissue extracts were harvested from the indicated group of mice and immunoblotted with specific antibodies. **h** WT and *Crbn* null mice were fed a CD and an HFD for 14 weeks. Gene expression was analyzed using qPCR with gene-specific primers. **i** Tissue extracts were analyzed by Western blot analysis with the indicated antibodies. **j** Measurement of blood glucose levels in the indicated mouse groups. *n* = 4-5 mice per group. ^*^P < 0.05, ^**^P < 0.01, and ^***^P < 0.001 vs. Ad-GFP, Fed infected Ad-Scram mice, CD-fed WT mice. ^#^*P* < 0.05 vs. Fast infected Ad-Scram mice, HFD-fed WT mice.

We sought to identify the critical role of CRBN in the regulation of hepatic gluconeogenesis with Ad-*Crbn* in mice. Ad-*Crbn* mice had substantially higher expression of the BTG2 and CREBH genes as well as blood glucose levels than Ad-GFP control mice but not the AMPK activity (Fig. 2a–c). Indeed, Ad-*Crbn*-infected mice showed significant impairment in glucose tolerance (Fig. 2d). Insulin intolerance was exacerbated by Ad-*Crbn*, indicating decreased insulin sensitivity by Ad-*Crbn* (Fig. 2e). Next, we investigated the impact of CRBN on gluconeogenic signals in Ad-sh*Crbn*-infected mice. The expression of the CRBN, BTG2, and CREBH genes was enhanced by fasting, and this phenomenon was disrupted when *Crbn* was silenced (Fig. 2f, g). As expected, an HFD increased the expression of these genes and blood glucose levels, while these phenomena were markedly attenuated in *Crbn* KO mice (Fig. 2h–j). Collectively, these observations indicate that CRBN functions as a key regulator of hepatic gluconeogenesis.

### BTG2 increases hepatic gluconeogenesis via the induction of *Crebh*

**Fig. 3:**
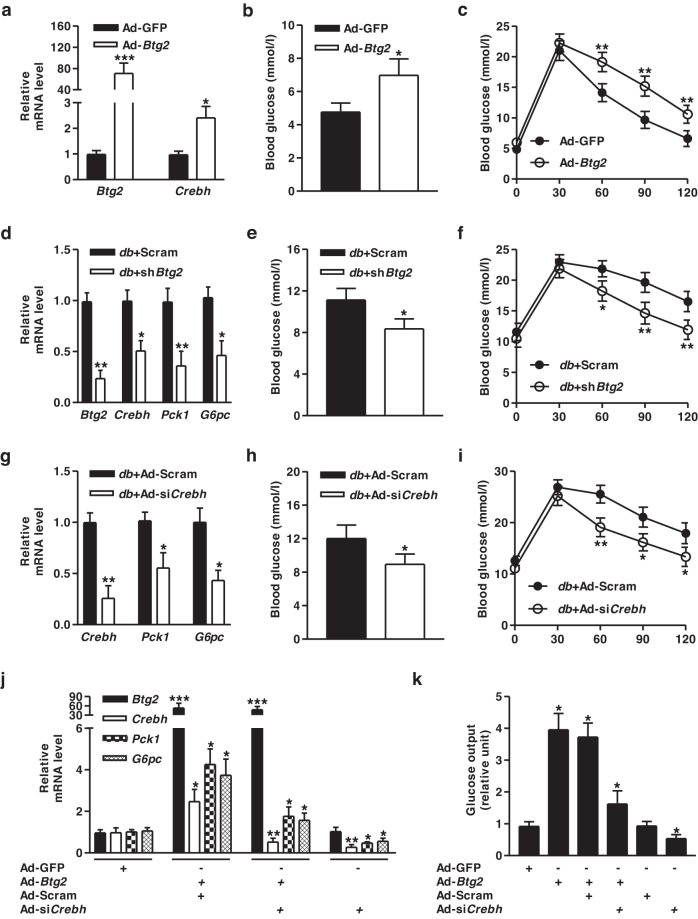
*Btg2* increases hepatic gluconeogenesis via the induction of *Crebh*. **a–c** WT mice were intravenously injected with Ad-GFP and Ad-*Btg2* for 10 days. **a** Gene expression analysis using gene-specific qPCR primers. **b** Blood glucose levels in these mice at Day 10 after adenoviral infection. **c** Glucose tolerance test in these mice at Day 4 after adenoviral infection. **d–f** *db*/*db* mice were injected with lentivirus-Scram and lentivirus-sh*Btg2* via the tail vein for 10 days. **d** Total RNA was utilized for qPCR analysis with gene-specific primers. **e** Blood glucose levels in these mice at Day 10 after lentiviral infection. **f** Glucose tolerance test in these mice at Day 4 after lentiviral infection. **g–i** *db*/*db* mice were injected with Ad-Scram and Ad-si*Crebh* via the tail vein for 10 days. **g** Total RNA was utilized for qPCR analysis with gene-specific primers. **h** Blood glucose levels in mice at Day 10 after adenoviral infection. **i** Glucose tolerance test in these mice at Day 4 after adenoviral infection. *n* = 4-5 mice per group. **j** Mouse primary hepatocytes were infected with Ad-GFP, Ad-Scram, Ad-*Btg2*, and Ad-si*Crebh* for 36 h and then analyzed using qPCR with various primers. **k** Glucose output assay under the indicated conditions. Data are representative of at least three independent experiments. ^*^P < 0.05, ^**^P < 0.01, and ^***^P < 0.001 vs. Ad-GFP, Ad-*Btg2*, or *db/db* mice.

Our previous study reported that gluconeogenic stimuli regulate the expression of BTG2 and its relevant target genes involved in hepatic glucose metabolism^19^. Thus, we hypothesized that BTG2 plays a crucial role in regulating hepatic gluconeogenesis in mice. We tested this hypothesis by infecting mice with Ad-*Btg2* and found significantly increased expression of *Crebh* mRNA and blood glucose levels (Fig. 3a, b). Furthermore, we performed glucose tolerance tests in Ad-*Btg2*-infected mice and found that glucose tolerance was markedly impaired in Ad-*Btg2*-infected mice compared to Ad-GFP control mice (Fig. 3c). To examine the role of *Btg2* in hepatic gluconeogenesis in diabetic mice, we administered sh*Btg2* to *db*/*db* mice. High basal levels of gluconeogenic genes, blood glucose, and glucose tolerance were reduced by disrupting *Btg2* expression in *db/db* mice (Fig. 3d–f). Likewise, Ad-si*Crebh-*infected *db*/*db* mice showed attenuated gluconeogenic genes, blood glucose, and glucose tolerance (Fig. 3g–i). We sought to investigate the direct effect of *Crebh* on *Btg2*-mediated hepatic gluconeogenesis. Overexpression of *Btg2* increased the expression of hepatic gluconeogenic enzyme genes and glucose production in primary hepatocytes (Fig. 3j, k), whereas these phenomena were prominently reduced by silencing of *Crebh*. Overall, these findings suggest that *Btg2* increases hepatic gluconeogenesis via the induction of *Crebh*.

### Melatonin ameliorates hepatic gluconeogenesis in diabetic mice

**Fig. 4:**
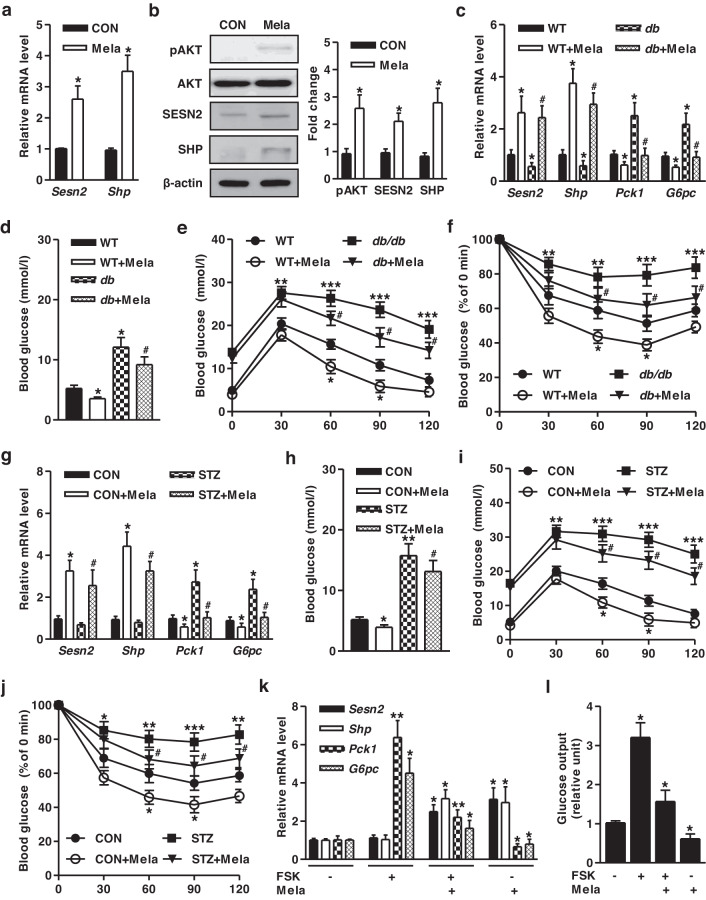
Melatonin ameliorates hepatic gluconeogenesis in diabetic mice. **a, b** Melatonin (20 mg/kg) was administered to WT mice once daily for 14 days. **a** Total RNA was utilized for qPCR analysis with gene-specific primers. **b** Protein levels in liver tissue extracts were analyzed by immunoblotting using specific antibodies. **c–f** WT and *db*/*db* mice were administered melatonin by daily oral gavage for 14 days. **c** Total RNA was utilized for qPCR analysis with gene-specific primers. **d** Blood glucose levels in the indicated groups on Day 14 in melatonin-fed mice. **e** Glucose tolerance test in mice at Day 8 after melatonin administration. **f** Insulin tolerance test in the indicated groups on Day 12 after melatonin treatment. **g–j** WT and STZ-induced diabetic mice were administered melatonin by daily oral gavage for 14 days. **g** Total RNA was utilized for qPCR analysis with gene-specific primers. **h** Blood glucose levels in these mice at Day 14 after melatonin administration. **i** Glucose tolerance test in these mice at Day 8 after melatonin administration. **j** Insulin tolerance test in these mice at Day 12 after melatonin treatment. *n* = 4-5 mice per group. **k, l** Mouse primary hepatocytes were treated with FSK (10 μM) and melatonin (500 μM) for 12 hr. **k** Total RNA was analyzed using qPCR with the indicated primers. **l** Glucose output assay in mouse primary hepatocytes exposed to FSK and melatonin for 12 h. Data are representative of at least three independent experiments. ^*^P < 0.05, ^**^P < 0.01, ^***^P < 0.001 vs. the untreated control, WT mice, FSK-treated cells. ^#^*P* < 0.05 vs. *db/db* mice, STZ mice.

We investigated the pivotal role of melatonin in glucose metabolism. Melatonin administration significantly increased the phosphorylation of AKT and the expression of the SESN2 and SHP genes in the liver and other tested tissues of mice but not in the lung (Fig. 4a, b, Supplementary Fig. 1). Melatonin-challenged WT and *db/db* mice exhibited diminished gluconeogenic gene expression and blood glucose levels by stimulating *Sesn2* and *Shp* expression compared to individual controls (Fig. 4c, d). Moreover, impaired glucose tolerance and insulin sensitivity in *db*/*db* mice were improved by melatonin (Fig. 4e, f). We observed a similar improvement pattern by melatonin in STZ-induced insulin-deficient mouse models. Melatonin administration to STZ-exposed mice resulted in decreased gluconeogenic gene expression and blood glucose levels by increasing *Sesn2* and *Shp* mRNA expression (Fig. 4g, h). Melatonin-exposed STZ mice had significantly better glucose tolerance and insulin sensitivity relative to individual controls (Fig. 4i, j). Next, we examined whether melatonin could inhibit gluconeogenic gene expression in primary hepatocytes. FSK treatment increased hepatic gluconeogenic genes and glucose output but not *Sesn2* and *Shp* expression. Conversely, melatonin repressed FSK-induced gluconeogenic genes and glucose production by upregulating *Sesn2* and *Shp* (Fig. 4k, l). Collectively, these data demonstrated that melatonin could decrease hepatic gluconeogenesis by inducing SESN2 and SHP expression in diabetic mouse models.

### Induction of SESN2 by melatonin inhibits hepatic glucose metabolism

**Fig. 5:**
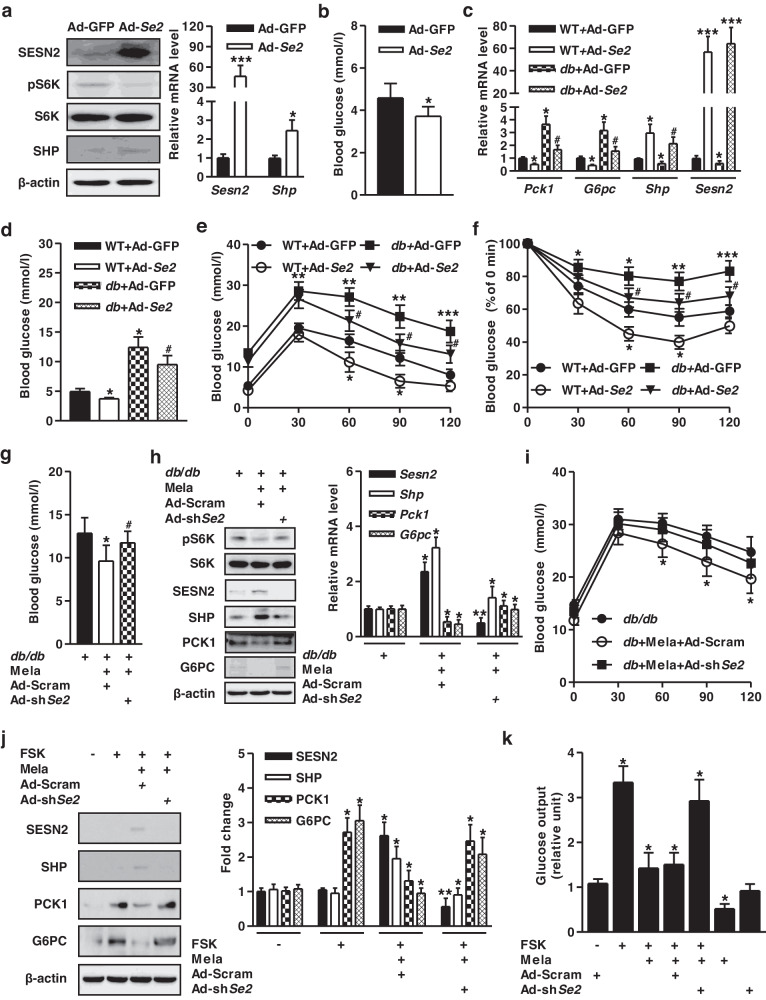
Induction of SESN2 by melatonin inhibits hepatic gluconeogenesis. **a, b** WT mice were injected with Ad-GFP and Ad-*Sesn2* (Ad-*Se2*) via the tail vein for 10 days. **a** Tissue extracts were harvested from the indicated group of mice and immunoblotted for the indicated proteins (*left panel*). Total RNA was utilized for qPCR analysis with gene-specific primers (*right panel*). **b** Blood glucose levels on Day 10 in Ad-*Sesn2*-infeced mice. **c–f** WT and *db/db* mice were injected with Ad-GFP and Ad-*Se2* for 10 days. **c** Total RNA was analyzed by qPCR with gene-specific primers. **d** Blood glucose levels in these mice at Day 10 after adenoviral infection. **e** Glucose tolerance test in mice at Day 4 after adenoviral infection. **f** Insulin tolerance test in mice at Day 8 after adenoviral transduction. **g–i** *db/db* mice were administered melatonin by daily oral gavage for 10 days and then intravenously infected with Ad-Scram and Ad-sh*Sesn2* (sh*Se2)* for 10 days. **g** Blood glucose level in the indicated groups on Day 10 after melatonin and adenoviral injection. **h** Various protein levels in liver tissue extracts were analyzed by immunoblotting using specific antibodies (*left panel*). Total RNA was utilized for qPCR analysis with gene-specific primers (*right panel*). **i** Glucose tolerance test in the indicated groups on Day 4 after melatonin and adenoviral injection. *n* = 4-5 mice per group. **j** Mouse primary hepatocytes were transduced with Ad-Scram and Ad-sh*Se2* for 36 h and then treated with FSK and melatonin for 12 h. Whole-cell extracts were isolated and analyzed using Western blot analysis with the indicated antibodies. **k** Glucose output assay in the indicated conditions of mouse primary hepatocytes. Data are representative of at least three independent experiments. ^*^P < 0.05, ^**^P < 0.01, ^***^P < 0.001 vs. the untreated control, Ad-GFP, Ad-Scram, and FSK-treated cells. ^#^*P* < 0.05 vs. *db/db*-infected Ad-GF*P* mice and *db/db*- and Mela-treated Ad-Scram mice.

To elucidate the potent role of SESN2 as a negative regulator of hepatic gluconeogenesis, we used Ad-*Sesn2* (Ad-*Se2*)-infected mice. Ad-*Se2* elevated *Shp* mRNA expression and reduced mechanistic target of rapamycin complex 1 (mTORC1)-dependent phosphorylation of ribosomal protein S6 kinase 1 (S6K), leading to decreased blood glucose levels compared to those of the Ad-GFP control groups (Fig. 5a, b). We investigated the crucial role of *Sesn2* in glucose metabolism in diabetic mice. Ad-*Se2*-infected WT and *db/db* mice exhibited decreased gluconeogenic genes and blood glucose levels by inducing *Shp* expression (Fig. 5c, d). Ad-*Se2*-infected *db*/*db* mice exhibited improved glucose and insulin tolerance relative to Ad-GFP-infected *db*/*db* mice (Fig. 5e, f). Melatonin-fed *db/db* mice showed attenuation of the phosphorylation status of S6K, gluconeogenic genes, and blood glucose levels, whereas these phenomena were reversed when *Sesn2* (*Se2*) was disrupted (Fig. 5g, h). However, melatonin-induced SESN2 and SHP gene expression was negated by silencing *Sesn2*, resulting in weak glucose tolerance by *Sesn2* knockdown in *db/db* mice (Fig. 5h, i). FSK-induced hepatic gluconeogenic genes and glucose production were repressed by melatonin, and this phenomenon was rescued by silencing *Sesn2*. Melatonin-induced SESN2 and SHP protein levels were abolished by silencing *Sesn2* (Fig. 5j, k). Taken together, these findings suggest that melatonin-induced *Sesn2* inhibits hepatic gluconeogenesis in diabetic mice and primary hepatocytes.

### Melatonin represses hepatic glucose metabolism by elevating SHP expression

**Fig. 6:**
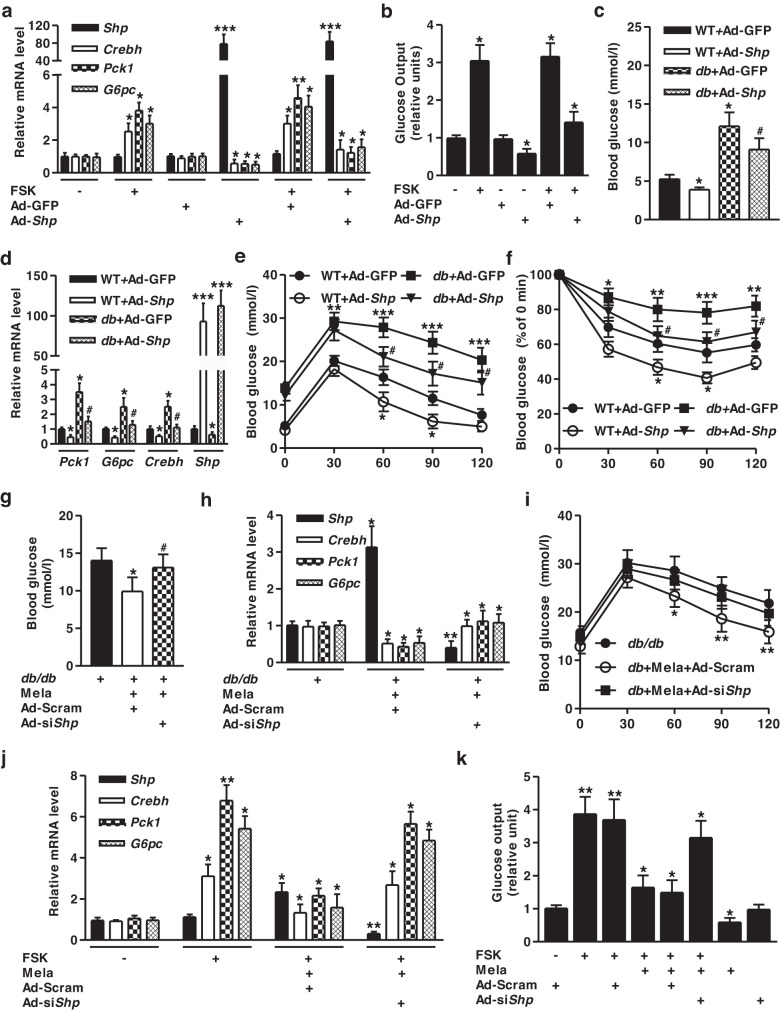
Melatonin represses hepatic gluconeogenesis by elevating SHP expression. **a, b** Mouse primary hepatocytes were infected with Ad-*Shp* for 36h and then treated with FSK for 12h. **a** Total RNA was analyzed using qPCR with the indicated primers. **b** Glucose output assay under the indicated conditions. **c–f** WT and *db/db* mice were infected with Ad-GFP and Ad-*Shp* via the tail vein for 10 days. **c** Blood glucose levels on Day 10 in Ad-*Shp*-transduced mice. **d** Gene expression analysis using gene-specific qPCR primers. **e** Glucose tolerance test in mice at Day 4 after adenoviral infection. **f** Insulin tolerance test in mice at Day 8 after adenoviral transduction. **g–i** *db/db* mice were administered melatonin by daily oral gavage for 10 days and then intravenously infected with Ad-Scram and Ad-si*Shp* for 10 days. **g** Blood glucose levels in the indicated groups on Day 10 after melatonin and adenoviral injection. **h** Total RNA was utilized for qPCR analysis with gene-specific primers. **i** Glucose tolerance test in the indicated groups on Day 4 after melatonin and adenoviral injection. *n* = 4-5 mice per group. **j, k** Mouse primary hepatocytes were infected with Ad-Scram and Ad-si*Shp* for 36 hr and then treated with FSK and melatonin for 12 h. **j** Gene expression was analyzed using qPCR with various primers. **k** Glucose output assay performed using samples from the indicated conditions. Data are representative of at least three independent experiments. ^*^P < 0.05, ^**^P < 0.01,^***^P < 0.001 vs. untreated control, Ad-GFP, Ad-Scram, and FSK-treated cells. ^#^*P* < 0.05 vs. *db/db*-infected Ad-GF*P* mice and *db/db*- and Mela-treated Ad-Scram mice.

To test the potential role of SHP in melatonin-mediated repression of gluconeogenesis, we examined the effect of SHP on the transcription of hepatic gluconeogenic genes and glucose output with Ad-*Shp*. FSK-induced increases in *Crebh* and gluconeogenic gene expression were attenuated by Ad-*Shp* (Fig. 6a). FSK-induced glucose production was markedly reduced by Ad-*Shp* (Fig. 6b). The *db*/*db* mice were utilized to evaluate the functional contribution of SHP to hepatic glucose metabolism in response to diabetic conditions. Ad-*Shp* decreased the elevated blood glucose levels and the expression of these genes in *db*/*db* mice compared to Ad-GFP-infected *db*/*db* mice (Fig. 6c, d). Interestingly, Ad-*Shp* improved glucose and insulin sensitivity by attenuating *db*/*db*-induced glucose and insulin intolerance (Fig. 6e, f). Melatonin reduced blood glucose levels, but this effect was reversed by *Shp* knockdown in *db/db* mice (Fig. 6g). Melatonin markedly decreased *Crebh*, *Pck1*, and *G6pc* mRNA expression by upregulating *Shp* expression, whereas this inhibitory effect of melatonin was recovered by silencing *Shp* (Fig. 6h), indicating exacerbated glucose intolerance by *Shp* knockdown (Fig. 6i). The increased expression of these genes and glucose production by FSK were diminished by melatonin, and this phenomenon was reversed by *Shp* knockdown (Fig. 6j, k). Overall, our findings demonstrated that SHP mediates the deteriorative effect of melatonin on hepatic gluconeogenesis under diabetic conditions.

### SHP inhibits hepatic gluconeogenesis via the competition of BTG2 and the interaction of CREBH

**Fig. 7:**
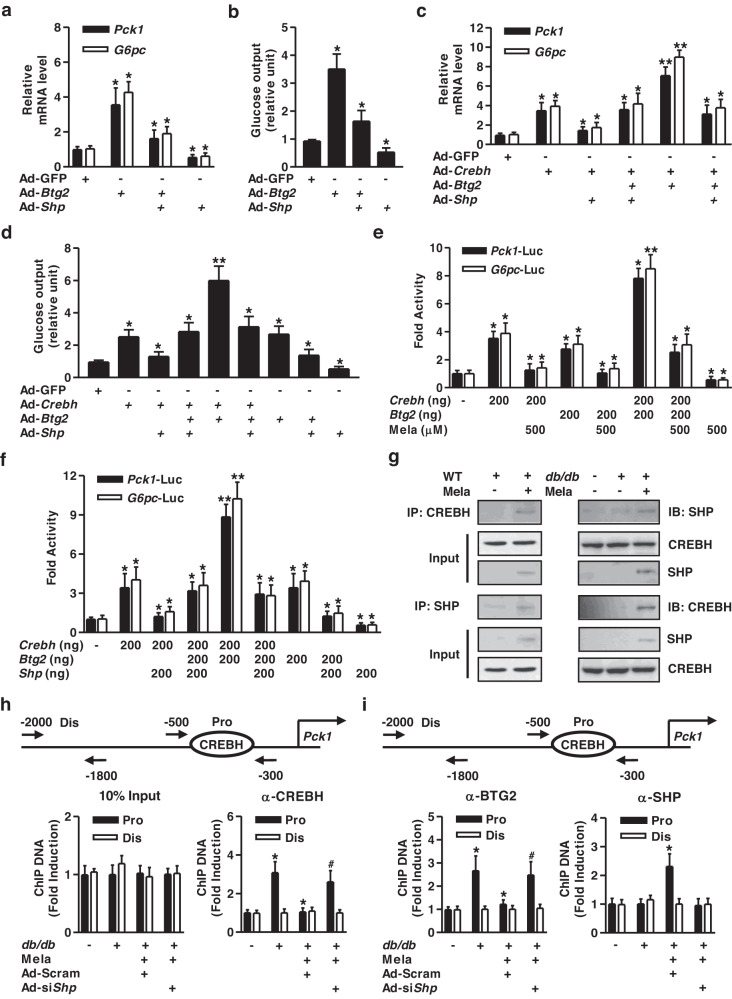
SHP inhibits hepatic gluconeogenesis by interfering with the BTG2 and CREBH interaction. **a, b** Mouse primary hepatocytes were infected with Ad-GFP, Ad-*Btg2*, and Ad-*Shp* for 36 h. **a** Gene expression analysis using gene-specific primers. **b** Glucose output assay under the indicated conditions. **c, d** Mouse primary hepatocytes were infected with Ad-GFP, Ad-*Crebh*, Ad-*Btg2*, and Ad-*Shp* for 36 h. **c** Gene expression was analyzed using qPCR with various primers. **d** Glucose output assay in the indicated conditions of mouse primary hepatocytes. ^*^P < 0.05, ^**^P < 0.01 vs. Ad-GFP, individual- or coinfected cells. **e** AML-12 cells were transiently transfected with *Crebh*, *Btg2*, and the indicated reporter genes for 36 hr. After transfection, cells were treated with melatonin for 12 h. **f** AML-12 cells were cotransfected with *Crebh*, *Btg2*, *Shp*, and the indicated reporter genes for 36 h. Luciferase activity was normalized to β-galactosidase activity to correct for transfection efficiency. Data are representative of at least three independent experiments. ^*^P < 0.05 vs. the untreated control, individual transfected or cotransfected cells. **g** Coimmunoprecipitation (IP) assays from liver extracts showed the functional interaction between CREBH and SHP under the observed conditions. Total protein extracts were utilized for immunoprecipitation and/or immunoblotting with specific antibodies. **h** ChIP assay for the occupancy of CREBH on the *Pck1* gene promoter. *db/db* mice were exposed to Mela and then infected with Ad-Scram and Ad-si*Shp* under the indicated conditions. Purified DNA samples were subjected to PCR using specific primers binding to the specific proximal (Pro) and nonspecific distal (Dis) regions on the *Pck1* gene promoter. Ten percent of the soluble chromatin was used as input. **i** ChIP assay showing the occupancy of BTG2 and SHP on the *Pck1* promoter under the indicated conditions. Soluble chromatin was immunoprecipitated with BTG2 and SHP antibodies. *n* = 4-5 mice per group. ^*^P < 0.05 vs. untreated control, *db/db* mice. ^#^*P* < 0.05 vs. *db/db*- and Mela-treated Ad-Scram mice.

To determine whether the SHP-mediated reduction in hepatic gluconeogenesis is mediated by the BTG2-CREBH axis, we examined the effect of SHP on BTG2- and CREBH-mediated hepatic gluconeogenic gene expression and glucose production in primary hepatocytes. Ad-*Btg2*-mediated increases in *Pck1* and *G6pc* expression and glucose production were reduced by Ad-*Shp* (Fig. 7a, b). Increased glucose production and expression of gluconeogenic genes by Ad-*Crebh* and/or Ad-*Btg2* were strikingly attenuated by Ad-*Shp* (Fig. 7c, d). We confirmed that melatonin regulates the transcriptional activity of gluconeogenic genes. *Pck1* and *G6pc* promoter activities were synergistically increased by *Crebh* and *Btg2* but not by melatonin treatment (Fig. 7e). We also verified the competition between *Btg2* and *Shp* for the transcriptional activity of *Crebh* on gluconeogenic gene promoters. Notably, *Btg2* increased and *Shp* repressed *Crebh*-mediated activation of gluconeogenic gene promoter activities. Cotransfection of *Shp* attenuated the synergistic activation of gluconeogenic promoter activities by *Btg2* and *Crebh* (Fig. 7f). We examined whether SHP modulates CREBH transcriptional activity via physical interaction. Thus, we performed co-IP assays to test the endogenous association between SHP and CREBH in WT and *db*/*db* mice. There was a clear increase in the association of SHP and CREBH in vivo in melatonin-exposed WT and *db*/*db* mice compared to their respective unexposed controls (Fig. 7g). These findings indicate that the transcriptional regulation of hepatic gluconeogenesis by *Btg2* and *Shp* may be accomplished by competition and direct interaction between these factors for the association with *Crebh* on gluconeogenic gene promoters.

Finally, we evaluated the effect of SHP on the recruitment of endogenous CREBH to the *Pck1* promoter. CREBH directly binds to the proximal region (Pro) of the *Pck1* promoter, and this interaction was negated in melatonin-exposed *db/db* mice. However, melatonin failed to abolish CREBH occupancy on the *Pck1* promoter by silencing *Shp* (Fig. 7h). To confirm whether BTG2 and SHP affect CREBH occupancy on the *Pck1* gene promoter, we performed a ChIP assay in *db/db* mice. As shown in Fig. 7i, induction of BTG2 in *db*/*db* mice and melatonin-induced SHP recruitment to the proximal region of the *Pck1* promoter were observed. Interestingly, the BTG2 increase in *db*/*db* mice promoted CREBH binding to the proximal region of the *Pck1* promoter, and this recruitment was completely ablated in melatonin-exposed mice. Conversely, melatonin-stimulated SHP binds to the BTG2-CREBH complex to inhibit its binding to the proximal region of the *Pck1* promoter. However, knockdown of *Shp* inhibited melatonin-mediated recruitment of CREBH to the proximal region of the *Pck1* promoter. Overall, these findings suggest that melatonin-induced SHP inhibits hepatic gluconeogenesis via the competition of BTG2 and the interaction of CREBH.

## Discussion

Our goal was to identify the novel molecular mechanism of melatonin in the regulation of hepatic gluconeogenesis. We established that gluconeogenic stimuli enhance hepatic gluconeogenesis mediated by the CRBN-BTG2-CREBH signaling pathway. Melatonin attenuates fasting- and diabetes-induced hepatic gluconeogenesis by stimulating the SESN2-SHP axis. Upregulation of SHP by melatonin repressed hepatic gluconeogenic gene transcription via the competition of BTG2 and the interaction of CREBH. Therefore, the melatonin-SESN2-SHP signaling network ameliorates hepatic glucose homeostasis by decreasing the CRBN-BTG2-CREBH axis in diabetic mice and primary hepatocytes. Based on these findings, we propose that the melatonin-SESN2-SHP signaling pathway may provide a novel molecular mechanism underlying the regulation of hepatic gluconeogenic gene expression and glucose metabolism, including glucose production, glucose excursion, and insulin sensitivity.

Lee et al. demonstrated that *Crbn* KO mice showed highly activated endogenous AMPK activity in diverse tissues^31^. HFD-fed *Crbn* KO mice showed lower expression of lipogenic genes and decreased gluconeogenesis, resulting in improved glucose intolerance and insulin resistance^15^. This study suggested that *Crbn* deficiency ameliorates lipogenesis and gluconeogenesis by stimulating AMPK and ACC activity in HFD-fed mice. Our current findings revealed that CRBN expression was increased during fasting, diabetes, and HFD and in DM patients (Fig. 1), and Ad-*Crbn* impaired glucose clearance and insulin sensitivity when compared to those of the Ad-GFP control groups. However, the disruption of *Crbn* resulted in decreased fasting-induced BTG2 and CREBH gene expression, and we also observed this phenomenon in *Crbn* KO mice (Fig. 2). These observations clearly suggested the functional role of CRBN in hepatic gluconeogenesis. Our observation implies that CRBN is highly expressed in diabetic mice and human patients with DM, and CRBN positively regulates hepatic glucose metabolism by stimulating BTG2 and CREBH as well as attenuating AMPK.

Next, we investigated the mechanism by which BTG2 induces CREBH in diabetic mice. Hepatic gluconeogenesis is mediated by transcriptional regulation of the CREB, PCK1, and G6PC genes via increased BTG2 expression, and CREBH has a crucial role in glucose and lipid metabolism in the liver and small intestine^18,21,32^. These results are consistent with our findings that Ad-*Btg2* increased *Crebh* gene expression and glucose production, resulting in impaired glucose tolerance (Fig. 3a–c), whereas *Btg2*- and *Crebh*-silenced *db*/*db* mice exhibited decreased gluconeogenic gene expression and reduced glucose production and glucose intolerance (Fig. 3d–i). Collectively, these results suggest that transcription of the *Pck1* and *G6pc* genes is enhanced by *Btg2* through the induction of *Crebh* in diabetic mice, which is a coregulator of BTG2 in the liver during diabetic conditions. Although *Pck1* and *G6pc* gene expression was strongly increased in Ad-*Btg2*-infected primary hepatocytes and mice, this phenomenon was negated by silencing *Crebh*. However, the regulatory mechanism of the BTG2-CREBH signaling pathway on hepatic gluconeogenesis has not been fully established. Further studies could identify a detailed underlying molecular mechanism by utilizing the hallmark of genetically engineered animal models, such as tissue-specific *Btg2* and *Crebh* knockout and knock-in mice.

Previous reports have shown that melatonin dysregulates hepatic gluconeogenesis by activating the SIRT1-STAT3 signaling pathway and hypothalamic PI3K-AKT axis in rats^9,10^. We hypothesized that the increase in SESN2 by melatonin regulates hepatic gluconeogenesis. This hypothesis raised intriguing questions regarding whether SESN2 functionally regulates hepatic gluconeogenic gene expression and glucose production. We found that melatonin challenge protects against hepatic gluconeogenesis by stimulating SESN2 and SHP expression in WT and diabetic mice (Fig. 4). Moreover, Ad-*Sesn2* and Ad-*Shp* reduced gluconeogenic gene expression, blood glucose, glucose excursion, and insulin sensitivity via the induction of SHP, whereas this phenomenon was ablated by *Sesn2* and *Shp* knockdown in diabetic mice (Figs. 5, 6). Therefore, melatonin-stimulated SESN2 may increase SHP gene expression in WT and diabetic mice, resulting in decreased hepatic gluconeogenesis and subsequently improved insulin resistance. Based on these results, we speculate that melatonin alleviates hepatic glucose metabolism via enhancement of the SESN2-SHP signaling pathway in response to diabetic conditions. Future studies may explain whether SESN2 also modulates the role of SHP in regulating hepatic gluconeogenesis in tissue-specific conditional knockout of the SESN2 gene.

Our previous studies have demonstrated that SHP alone or metformin-induced SHP represses gluconeogenic signal- and growth hormone-induced PCK1/G6PC gene expression^33–35^. However, the exact molecular mechanism linking SHP and CREBH in diabetes-mediated hepatic gluconeogenesis remains unknown. Based on these findings, we suggest that the melatonin-SHP signaling pathway can explain the molecular mechanism of diabetes-induced gluconeogenic gene transcription. First, we demonstrated that melatonin and SHP attenuate the induction of gluconeogenic gene promoter activities by CREBH and BTG2 (Fig. 7e, f). Second, melatonin and SHP decreased gluconeogenic gene transcription by physical association of SHP with CREBH (Fig. 7g). Third, diabetes-stimulated CREBH recruitment to the *Pck1* gene promoter was diminished in melatonin-fed mice but was significantly restored by silencing *Shp* (Fig. 7h). Finally, melatonin-induced SHP binds to the BTG2-CREBH complex to repress its recruitment to the *Pck1* promoter, whereas this phenomenon was rescued by *Shp* knockdown (Fig. 7i). Our current findings strongly validated a novel molecular mechanism between the melatonin-SHP signaling pathway and diabetic hepatic gluconeogenesis. However, further detailed studies are required to identify more novel molecular networks linked with melatonin-induced SHP expression and hepatic gluconeogenic gene transcription under diabetic conditions by modulating several unknown events, including chromatin remodeling, protein degradation, and microRNAs.

**Fig. 8:**
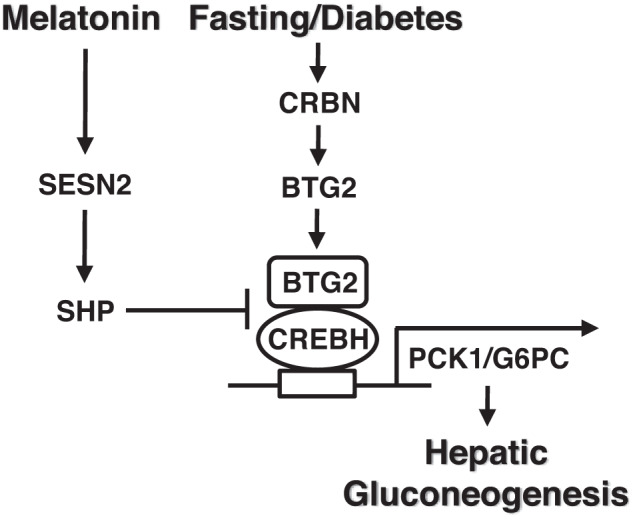
Regulation of hepatic gluconeogenesis by gluconeogenic signals and melatonin. Gluconeogenic signals (forskolin and fasting) and diabetes increase hepatic gluconeogenesis by upregulating the CRBN-BTG2-CREBH axis. Melatonin attenuates hepatic gluconeogenesis by stimulating the SESN2-SHP signaling pathway in diabetic mice and primary hepatocytes.

In conclusion, our current study suggests that gluconeogenic stimuli and diabetes deteriorate hepatic gluconeogenesis through the CRBN-BTG2-CREBH axis. Melatonin ameliorates hepatic glucose metabolism by enhancing the SESN2-SHP signaling pathway, while this protective effect of melatonin was negated by silencing *Sesn2* and *Shp* during fasting and diabetic conditions. We propose that the melatonin-SESN2-SHP axis protects against glucose homeostasis by inhibiting the CRBN-BTG2-CREBH signaling pathway under fasting and diabetic conditions, as described in Fig. 8. Our current findings provide the fundamental framework for a therapeutic strategy for the prevention of metabolic dysfunction in response to melatonin and a potential therapeutic approach for the treatment of metabolic diseases such as diabetes.

## Supplementary information

Supporting Information
