# Supplementary material for: Enhancement of the SESN2-SHP cascade by melatonin ameliorates hepatic gluconeogenesis by inhibiting the CRBN-BTG2-CREBH signaling pathway
Source: Exp Mol Med. 2023 Jul 24;55(7):1556–69. doi: 10.1038/s12276-023-01040-x (PMC10393991; doi:10.1038/s12276-023-01040-x)

Supplementary Fig. 1 Expression of *Sesn2* and *Shp* mRNA in liver and other tissues of mice. a-b WT mice were administered with melatonin by daily oral gavage for 14 days. Total RNAs were isolated from various tissues and utilized for qPCR analysis with gene-specific primers. $n = 5$ mice per group. *$P < 0.05$ vs. untreated control (CON).

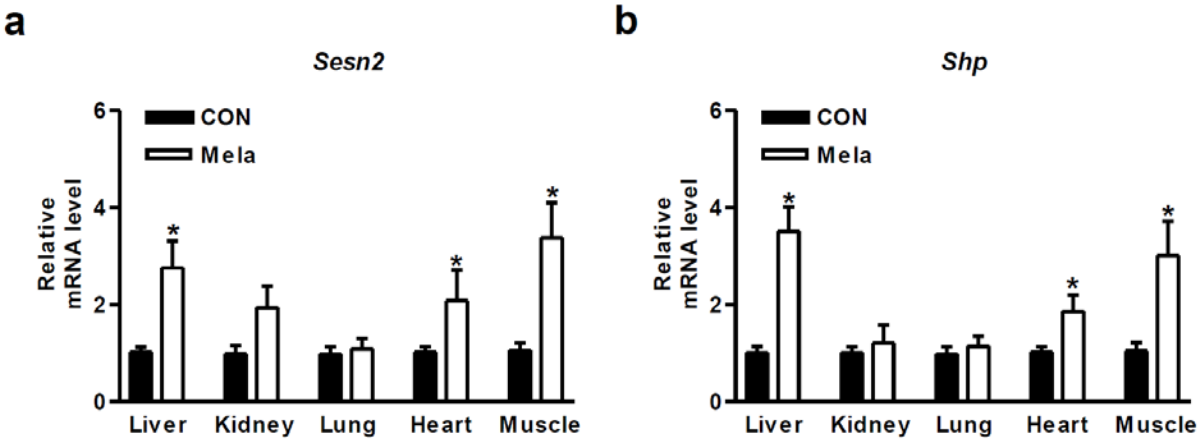

Supplement: Supplementary file 1 — Supporting Information [file 12276_2023_1040_MOESM1_ESM.pdf]
